# Retraction: NR2F1-AS1/miR-190a/PHLDB2 induces the epithelial–mesenchymal transformation process in gastric cancer by promoting phosphorylation of AKT3

**DOI:** 10.3389/fcell.2024.1491753

**Published:** 2024-09-11

**Authors:** Frontiers Editorial Office

The journal retracts the 22 October 2021 article cited above.

Following publication, Frontiers was alerted to the presence of non-verifiable cell line identifiers in this publication. Upon further examination, it was determined that core findings of this study were obtained using non-verifiable cell lines and/or cell lines known to be cross-contaminated with HeLa. This contamination compromises the data and conclusions of the article and the article has been retracted.

This retraction was approved by the Chief Executive Editor of Frontiers. The authors received a communication regarding the retraction and have not responded to the retraction. This communication has been recorded by the publisher.

